# An approach for prioritizing candidate genes from RNA-seq using preclinical cocaine self-administration datasets as a test case

**DOI:** 10.1093/g3journal/jkad143

**Published:** 2023-07-12

**Authors:** Annika Vannan, Michela Dell’Orco, Nora I Perrone-Bizzozero, Janet L Neisewander, Melissa A Wilson

**Affiliations:** School of Life Sciences, Arizona State University, Tempe, AZ 85287-4501, USA; Department of Neurosciences, University of New Mexico Health Science Center, University of New Mexico, Albuquerque, NM 87131-0001, USA; Department of Neurosciences, University of New Mexico Health Science Center, University of New Mexico, Albuquerque, NM 87131-0001, USA; School of Life Sciences, Arizona State University, Tempe, AZ 85287-4501, USA; School of Life Sciences, Arizona State University, Tempe, AZ 85287-4501, USA; Center for Evolution and Medicine, Arizona State University, Tempe, AZ 85287-4501, USA

**Keywords:** differential expression analysis, experimental design, animal model, drug seeking behavior, translational value, brain gene expression, evolutionary conservation, addiction, bioinformatics

## Abstract

RNA-sequencing (RNA-seq) technology has led to a surge of neuroscience research using animal models to probe the complex molecular mechanisms underlying brain function and behavior, including substance use disorders. However, findings from rodent studies often fail to be translated into clinical treatments. Here, we developed a novel pipeline for narrowing candidate genes from preclinical studies by translational potential and demonstrated its utility in 2 RNA-seq studies of rodent self-administration. This pipeline uses evolutionary conservation and preferential expression of genes across brain tissues to prioritize candidate genes, increasing the translational utility of RNA-seq in model organisms. Initially, we demonstrate the utility of our prioritization pipeline using an uncorrected *P*-value. However, we found no differentially expressed genes in either dataset after correcting for multiple testing with false discovery rate (FDR < 0.05 or <0.1). This is likely due to low statistical power that is common across rodent behavioral studies, and, therefore, we additionally illustrate the use of our pipeline on a third dataset with differentially expressed genes corrected for multiple testing (FDR < 0.05). We also advocate for improved RNA-seq data collection, statistical testing, and metadata reporting that will bolster the field's ability to identify reliable candidate genes and improve the translational value of bioinformatics in rodent research.

## Introduction

RNA-sequencing (RNA-seq) allows researchers to probe the genetic underpinnings of neurological disorders on a large scale. RNA-seq can be used to assess gene expression across the entire transcriptome, and thus this technique has the potential to reveal novel genetic targets for complex diseases. Many disorders are driven by gene-environment interactions which manifest in complex behavioral phenotypes that are difficult to disentangle in clinical populations (Goldman *et al.* 2005; Buckland 2008; Bray and O’Donovan 2019). However, few studies of neuropsychiatric disorders in human subjects are able to incorporate both behavioral information and an unbiased evaluation of genetic data, e.g. through RNA-seq or other genome wide-techniques (though see Marees *et al.* 2020). Because of this, preclinical animal models have been critical to our understanding of the behavioral and genetic correlates of disease. Animal models continue to provide a rich foundation for genetic research in the RNA-seq era, and the number of publicly available preclinical RNA-seq datasets is rapidly increasing. However, given the size and scope of transcriptome-wide gene expression datasets, there is a great need to improve candidate gene searches from preclinical RNA-seq data.

An important consideration for any preclinical study is translation to the human condition. For behavioral neuroscience datasets, translational utility relies not just on behavioral and psychological similarity between humans and animals, but also on convergent neurobiology. While neural circuits such as the reward system are broadly shared between mammals (Panksepp *et al.* 2002; Scaplen and Kaun 2016; Martínez-García and Lanuza 2018), gene expression variation between humans and rodents (e.g. throughout development (Cardoso-Moreira *et al.* 2019; Cardoso-Moreira *et al.* 2020)) may have implications for generating treatments based on rodent data. Some researchers have begun to tackle this issue by leveraging genome- and transcriptome-wide data across rodents and humans to identify convergent pathways and genes that may lead to more successful therapeutic targets or by conducting RNA-seq studies on both human and rodent tissue in tandem (Ray *et al.* 2018; Forero and González-Giraldo 2020; Huggett *et al.* 2020; Huggett and Stallings 2020; Olislagers *et al.* 2022). Another difficulty in selecting targets is that treatments for neurological disorders that are given systemically will affect other organs as well as the brain. Neurobiological mechanisms also vary across brain regions, such that targeting a gene or pathway in some areas may have unintended side effects for others. For example, treatments that target the dopamine system may affect reward and motivation through the nucleus accumbens (NAc) but incur movement-related side effects via the caudate and putamen (Iversen and Iversen 2007; Neisewander *et al.* 2014). Incorporating the general context of gene expression across the brain and body is another important consideration when choosing next steps after an initial RNA-seq analysis. This is true both for follow-up studies in rodents, such as functional validations of candidate genes and preclinical testing of novel therapeutic interventions and for long-term goals of translating findings to human patients.

Here, we utilized RNA-seq datasets from preclinical animal models of substance use disorders (SUDs) to develop and implement a pipeline for prioritizing candidate genes based on translational value. Specifically, we selected 2 publicly available RNA-seq datasets from cocaine self-administration (SA) studies in mice (Walker *et al.* 2018; Carpenter *et al.* 2020). Although these studies had methodological variation, they both featured a forced abstinence (ABS) period (no exposure to drug or the SA environment), which is a manipulation used preclinically to increase motivation for cocaine (Neisewander *et al.* 2000). Using the individual differentially expressed genes (DEGs) from each dataset (*P* < 0.05), we demonstrate the utility of considering evolutionary conservation and brain expression data when selecting candidate genes for further study through a novel pipeline. However, using a more stringent statistical threshold (false discovery rate (FDR) < 0.05 or 0.1), we found no significant DEGs in either dataset. Additionally, we compared results from our prioritization pipeline using the 2 prolonged ABS datasets with a set of DEGs (FDR < 0.05) from a third RNA-seq study in mice with a short ABS period (24 hr) after cocaine SA (Engeln *et al.* 2022). In addition our prioritization pipeline, we suggest that more robust experimental designs and statistical analysis will help improve the field's ability to use RNA-seq data to its fullest potential and allow researchers to leverage a collective knowledge to advance neuroscience and SUDs research.

## Materials and methods

### Dataset selection and availability

In rodent models of SUDs, cocaine SA is a learning process that primes the brain for motivation for drug in response to environmental or internal cues that invoke memory of the cocaine experience. This primed state intensifies over the course of ABS, as demonstrated in both rats (Tran-Nguyen *et al.* 1998; Neisewander *et al.* 2000; Grimm *et al.* 2001) and mice (Mead *et al.* 2007; Halbout *et al.* 2014; Alegre-Zurano *et al.* 2022), and is translationally relevant to humans (Gawin and Kleber 1986). We selected 2 publicly available RNA-seq datasets from male mice that included treatment groups relevant to cocaine motivation and ABS (Walker *et al.* 2018; Carpenter *et al.* 2020). For both studies, RNA-seq was performed on the NAc, a mesolimbic region fundamental to drug motivation and reward (Floresco 2015), and included groups of mice that underwent cocaine SA and forced ABS for a short (1 d) or long (28–30 d) interval. Because the datasets have been published previously, we hereafter refer to them by their first authors (Carpenter and Walker). Raw RNA-seq files for each study were downloaded from NCBI's Sequencing Read Archive (Carpenter: SRP234876, Walker: SRP132477) and reprocessed as described below to facilitate direct comparisons between the 2 datasets. Separately, we demonstrated our pipeline on a set of DEGs (FDR < 0.05) from another study of cocaine SA in male mice without reprocessing the RNA-seq data. This dataset from Engeln *et al.* (2022) utilized tissue from mice collected 24 hr after cocaine or saline SA and was selected due to a paucity of well-powered RNA-seq data from animals undergoing prolonged ABS. The list of DEGs was obtained directly from the original publication's supplement.

Summaries of relevant design parameters, including noteworthy differences between studies, along with descriptions of the treatment groups included in the present analysis, are given in Table 1. Additional information about files, detailed descriptions of analyses, and scripts are provided on GitHub (https://github.com/SexChrLab/RodentAddiction). This includes a script for users to run the prioritization pipeline on their own set of genes from either mouse or rat.

**Table 1.** Description of cocaine SA paradigms used in each study.

Study	Species	Sex	Drug	SA sessions	Schedule(s) of reinforcement	ABS	Postabstinence	Treatment groups in present analysis*^a^*	Sample size
Carpenter *et al.* (2020)	Mouse	Male	Cocaine (0.7 mg/kg) or Saline, IV	2 hr/d, 21d	FR1	1 or 28 d	Extracted whole NAc*^a^*	S28: Saline SA, 28d ABS C28: Cocaine SA, 28d ABS	6/group
Walker *et al.* (2018)	Mouse	Male	Cocaine (0.5 mg/kg) or Saline, IV	2 hr/d, 10–15d	Progression from FR1 up to FR2	1 or 30 d	1d ABS: None 30d ABS: Saline or Cocaine challenge injection and SA context re-exposure Extracted whole NAc*^a^*	S30: Saline SA, 30d ABS, Saline challenge injection and context re-exposure C30: Cocaine SA, 30d ABS, Saline challenge injection and context re-exposure	5–8/group
Engeln *et al.* (2022) *^b^*	Mouse	Male	Cocaine (0.5 mg/kg) or Saline, IV	2 hr/d, 10d	FR1	24 hr	Extracted VP	S1: Saline SA, 1d ABS C1: Cocaine SA, 1d ABS	4/group

Other tissues were extracted for these experiments, but only the NAc were included in this analysis.

The Engeln RNA-seq dataset was not reprocessed for the present study and was analyzed separately from the Carpenter and Walker datasets.

### RNA-seq processing and differential expression analysis

The datasets were reprocessed using the same methodology to allow for accurate comparisons between them. The project workflow from downloading sequencing files to obtaining DEGs is described in Supplementary Figure 1. Original sequencing files were assessed for quality using FastQC (Andrews 2010) to generate reports and MultiQC (Ewels *et al.* 2016) to visualize reports in aggregate. BBDuk (part of the BBTools suite; http://jgi.doe.gov/data-and-tools/bbtools) was used to trim reads from all experiments using the same basic parameters (“ktrim = r k = 21 mink = 11 hdist = 2 qtrim = rl trimq = 30 maq = 20”). The minlen parameter was set to half of the original read length, which varied between experiments. Adapters and overrepresented sequences were additionally trimmed where applicable. Before alignment, library type (e.g. unstranded and reverse-stranded) was determined with Salmon using the Ensembl GRCm39 mouse transcriptome index from Refgenie (Patro *et al.* 2015; Stolarczyk *et al.* 2020). A HISAT2 (Kim *et al.* 2019) genome index was created, and then trimmed reads were aligned using the library type identified by Salmon for the –rna-strandness parameter and other settings at default. We utilized HISAT2 for the final alignment because it is splice-aware, though other alignment tools are likely to give similar results (Srivastava *et al.* 2020). Gene read counts were quantified for primary alignments (−primary) with featureCounts (Liao *et al.* 2014) using the -s parameter to specify library type.

For each dataset, edgeR (Robinson *et al.* 2010) was used to transform and normalize read counts (e.g. from raw counts to fragments per kilobase per million mapped reads (FPKM)) and to filter out lowly expressed genes. Genes were retained if their mean expression was greater than 0.5 FPKM for at least one of the 2 treatment groups in a study and had a raw read count of at least 6 greater than or equal to the lowest sample size for that comparison. Raw counts were transformed to log_2_ counts per million (CPM), and multidimensional scaling (MDS) plots of the top 100 genes with the highest variance shared between groups were used to identify potential outlier samples. Genes were filtered and transformed again after outlier removal, and gene expression values from the remaining samples were normalized using the trimmed-means method (TMM) (Robinson and Oshlack 2010). Next, limma voom (Law *et al.* 2014; Ritchie *et al.* 2015) was used to build linear models that accounted for technical sources of variation (sequencing lane or batch) identified by the package variancePartition (Robinson *et al.* 2010). Linear models were fitted using the least squares method, and empirical Bayes statistics were generated for each contrast. DEGs for each study were determined using 3 statistical thresholds (FDR < 0.05, FDR < 0.1, and uncorrected *P-*value < 0.05).

### Development and assessment of a novel pipeline for narrowing candidate genes by translational potential

**Fig. 1.**
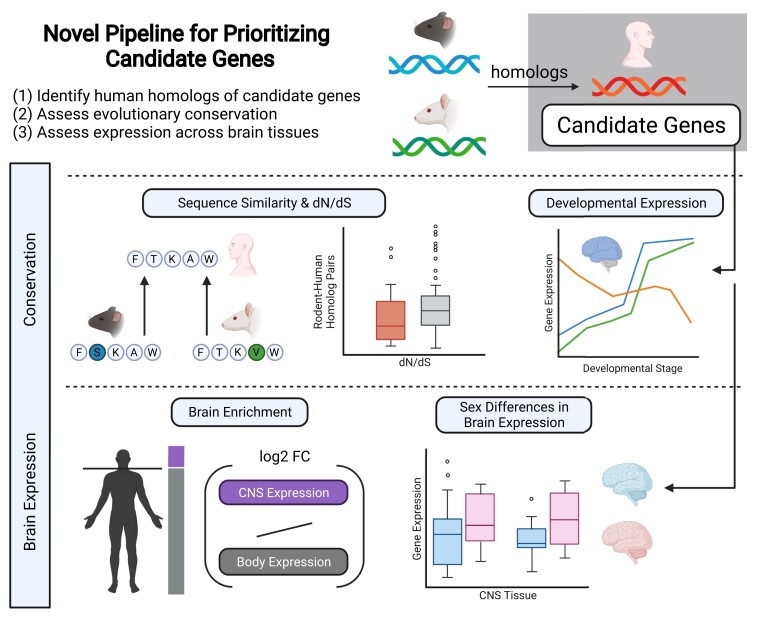
Novel pipeline for prioritizing candidate genes from RNA-seq data. After obtaining DEGs, genes can be narrowed based on translational potential by incorporating evolutionary conservation and brain expression data. Human orthologs of DEGs from rodent studies can be assessed with these criteria. For example, we utilize the DEGs from each dataset as “Candidate” genes in this pipeline. Candidate genes were analyzed for conservation across species by comparing sequence similarity, dN/dS, and developmental expression in the forebrain between HMR. Sequence similarity and dN/dS data were obtained from Ensembl through the R package biomaRt. Developmental expression data were obtained from Cardoso-Moreira *et al.* (2020). Next, Candidate genes were assessed for their specificity to the brain and expression across brain tissues, including possible sex differences, using human data from the Gene-Tissue Expression (GTEx) database.

Next, we implemented a pipeline aimed to improve preclinical RNA-seq analysis by focusing on the translational potential of DEGs in humans (Fig. 1). For this purpose, we analyzed the DEGs in each dataset (*P* < 0.05), as theoretical test cases to apply a novel pipeline that prioritized candidate genes based on evolutionary conservation and healthy human brain expression data. Though DEGs discovered from rodent studies do not need to be orthologous in human to be relevant to SUDs, for this translation-focused pipeline we utilized only those DEGs that had human orthologs. We obtained orthologs for the DEGs by utilizing biomaRt (Durinck *et al.* 2005, 2009) and comparing them to the rat genome Rnor_6.0 or human genome GRCh38.p13 (Ensembl release 106, April 2022). Orthologous human genes were considered theoretical “Candidate” genes that were implemented in the pipeline for each dataset. Some discrepancies exist in regards to human IDs and ortholog identification between Ensembl versions; see Supplementary File 1 for more information.

### Incorporating evolutionary conservation data to prioritize candidate genes

A primary goal of rodent research is to translate findings into human therapeutics, which relies on conservation of genetic and molecular mechanisms between species. We, therefore, sought to determine the evolutionary conservation of Candidate genes between human and mouse (HM). Additionally, we include comparisons to rat, as utilizing data from both of the most commonly studied animal models in SUDs may be informative to researchers that use both rodent species, for example by verifying evolutionary conservation of candidate genes obtained from mouse data before proceeding with studies of that gene in rat. We incorporated developmental gene expression data, sequence similarity, and the ratio of divergence at nonsynonymous and synonymous sites (dN/dS), which has been used as an indicator of evolutionary selective pressure acting on protein-coding genes (Yang and Bielawski 2000). For sequence similarity and dN/dS comparisons, Candidate genes were analyzed separately for mouse–human and rat–human orthologs, using only one-to-one orthologs. Sequence similarity scored as a percentage of the matched protein sequence (amino acids) of a rodent gene compared to its human ortholog, was obtained from the Ensembl database using biomaRt (Durinck *et al.* 2005, 2009). To obtain dN and dS values, an older Ensembl release (99; January 2020) was used due to availability of these metrics. As part of a comprehensive project on species differences in developmental gene expression, Cardoso-Moreira *et al*. (2020) utilized previously collected RNA-seq data from several tissues of human, mouse, rat, and other species across developmental time points (Cardoso-Moreira *et al.* 2019) to determine which genes diverged in their temporal expression patterns across species. Utilizing their data from the forebrain, a large region of the brain that includes the NAc and ventral pallidum (VP), we categorized Candidate genes as developmentally conserved between human, mouse, and rat (HMR), only HM, only human and rat (HR), or not conserved between humans and either rodent species (H).

### Incorporating brain specificity and expression data across central nervous system tissues to prioritize candidate genes

Human orthologs for the theoretical Candidate genes were examined for expression in healthy human tissues using the publicly available Genotype-Tissue Expression (GTEx) Project database (GTEx Consortium 2013; Melé *et al.* 2015) (https://www.gtexportal.org/home/). Expression data in transcripts per million (TPM) were obtained for 51 human tissues. This included 12 central nervous system (CNS) tissues, of which 10 were reward-related (amygdala, anterior cingulate cortex BA24, caudate, frontal cortex BA9, hippocampus, hypothalamus, NAc, putamen, pituitary gland, substantia nigra), and 2 were primarily related to motor function (spinal cord cervical C1, cerebellum). All CNS tissues were collected by the Miami Brain Bank, with the exception of 2 cortical and cerebellar tissues that were excluded from the present analysis, as they were replicates not collected by the same research group.

For each gene, the mean expression in TPM across individuals was summed for both CNS and non-CNS tissues, excluding brain replicates and cell lines. From this, a brain specificity score was calculated as the log_2_ fold change (FC) of the ratio of total CNS expression over total expression in other tissues. Candidate genes in each dataset were compared to all other genes not in any dataset using Mann–Whitney U tests with Bonferroni corrections. Though the starting datasets utilized male mice only, it is crucial to consider how expression of genes of interest might lose or gain translational potential if they are expressed in a sexually dimorphic manner. Thus, to assess for potential sex differences in CNS expression of human orthologs of Candidate genes, mean and median gene expression was compared between males and females. We focused only on samples that were age-matched between the sexes for individuals ≥ 55 years of age (i.e. a typical postmenopause age in women). This age group was selected to control for the effects of sex hormones on brain gene expression. However, since SUDs often arise during adolescence, researchers interested in understanding sex differences in gene expression in a younger age group may modify the pipeline to focus on 20–29 years of age, the youngest GTEx age group.

### Statistical analyses for the prioritization pipeline

Statistical analyses were performed in R. Candidate genes from each dataset were compared to all other genes not in either dataset using Mann–Whitney U tests with Bonferroni corrections (sequence similarity, dN/dS, brain specificity) or Fisher's exact tests (developmental conservation.) For general tissue expression, 2-way Type III ANOVAs were used to analyze effects of tissue and sex as well as Sex:Tissue interactions. Post hoc Tukey's tests were used to further assess the main effects or interactions. Fisher's exact tests were also used to compare the distribution of priority scores between datasets, with simulated *P-*values for large contingency tables.

### RNA-seq power analysis

Because only 1 DEG remained in any dataset after FDR correction (FDR < 0.05 or FDR < 0.1), we performed power analyses for each dataset. After filtering genes and removing outlier samples according to the above criteria, power analyses were conducted with the estimate of 0.8 power using a threshold of FDR < 0.1. Using the R package *ssizeRNA* (Bi and Liu 2016), the sample size required to achieve appropriate power was calculated based on values from each dataset, including the total number of genes (nGenes), the mean read count of the control group (mu), and tagwise dispersions estimated by edgeR (disp). Several values were used for the expected proportion of DEGs (0.05, 0.1, 0.2) and FC for each DEG (1.15, 1.25, 1.5, and 2).

## Results

### Differential expression analysis reveals discrepant results based on statistical thresholding

After filtering out genes with low expression, the datasets contained 10,026 (Carpenter) and 9,948 (Walker) genes each. Summaries of the processing for each sample and final statistics for each dataset are located in Supplementary Tables 1 and 2, respectively. For the Walker dataset, MDS, and variancePartition plots showed that the experimental variable sequencing batch explained a larger portion of the variation in gene expression than the treatment groups, so it was incorporated into the linear model before differential expression analysis. Supplementary Figures 2 and 3 contain MDS and variancePartition plots, along with voom (Law *et al.* 2014) results before and after linear modeling. No outlier samples were detected in either dataset, as all samples clustered near each other on the MDS plots. Final sample sizes were 12 (6/group; Carpenter) and 11 (5–6/group; Walker).

**Fig. 2.**
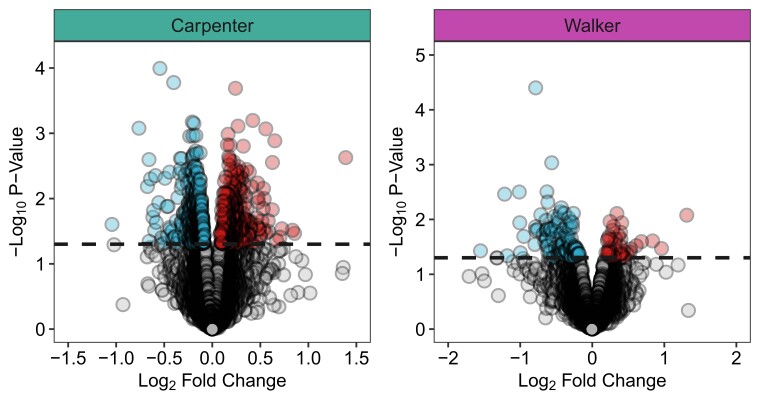
DEGs in the carpenter and walker datasets. Volcano plots depicting the spread of gene expression data for the Carpenter and Walker comparisons. Dashed lines indicate an uncorrected *P-*value cutoff of < 0.05 (horizontal). Genes that met this criterion are colored to indicate that they are downregulated (blue; left in each panel) or upregulated (red; right in each panel) in the prolonged ABS group relative to the short-term ABS group of a given dataset.

DEGs were assessed using 3 different statistical thresholds: FDR < 0.05, FDR < 0.1, and uncorrected *P* < 0.05) (Supplementary Tables 3 and 4). No DEGs were identified in either dataset with either FDR threshold. For the purposes of exploring our prioritization approach, we applied a nominal *P-*value threshold (Fig. 2). For the Carpenter contrast, there were 633 DEGs, with 304 up- and 329 downregulated in C28 relative to S28. For Walker, there were 139 DEGs, 39 up- and 100 downregulated in C30 relative to S30). The 2 datasets had 15 DEGs in common (Supplementary Table 5). Of these, 10 were regulated in the same direction, e.g. upregulated in the cocaine ABS group for both datasets. This included *Amz1*, *Btg1*, *Cacybp*, *Cartpt*, *Ccdc88c*, *Fabp7*, *Gm44266*, *Hspa8*, *Phlda1*, and *Pitpnm3* (shared and regulated in the same direction) as well as *B2m*, *Clec2d*, *Ifitm3*, *Ly6c1*, and *H2-Q4* (shared but with different directions of regulation). Human orthologs of the DEGs (*P* < 0.05) in each dataset (585 and 116 human genes respectively for Carpenter and Walker) were used as Candidate genes to demonstrate a novel pipeline for prioritizing candidate genes with translational potential by incorporating evolutionary conservation and healthy human brain expression data. However, given the low sample sizes and low power in each dataset as described later in the Results, the DEGs identified with a nominal *P*-value are not robust and require further validation. As such, the Carpenter and Walker Candidate genes should be taken as demonstration of the novel prioritization pipeline rather than specific genes of interest. For full names of all human genes discussed in the main text, see Supplementary File 1.

### Prioritizing candidate genes using evolutionary conservation

**Fig. 3.**
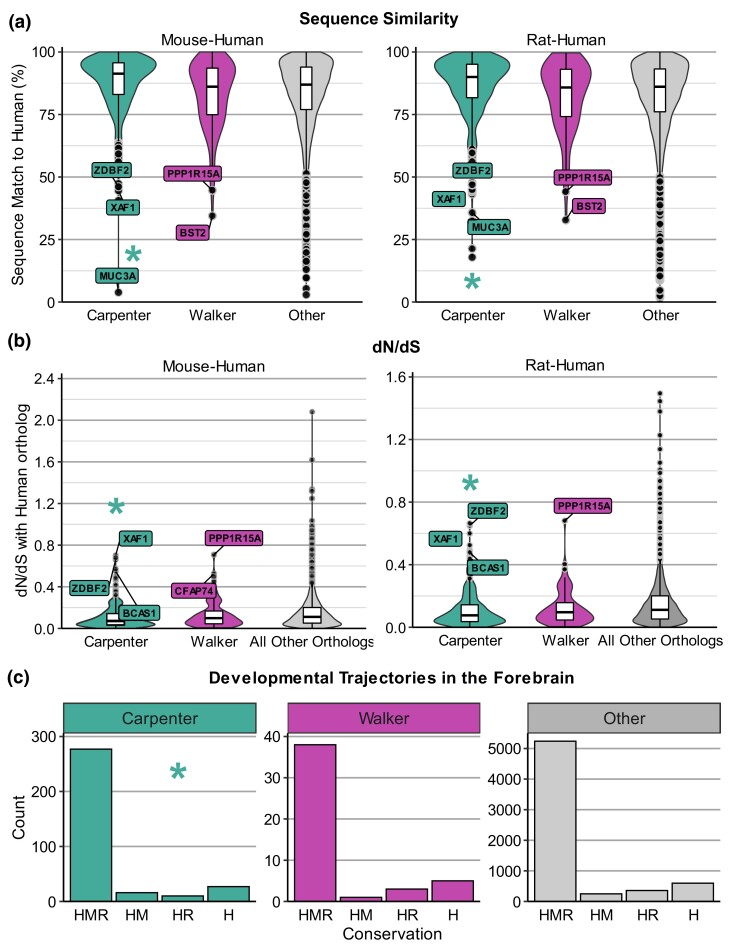
Conservation of candidate genes. a) Sequence similarity, measured as percentage similarity of the rodent gene to its human ortholog. b) dN/dS values for mouse–human and rat–human orthologs. c) Conservation of gene expression patterns across development in the forebrain, utilizing data from Cardoso-Moreira *et al.* 2020. HMR = conserved expression across development in HMR; HM = conserved expression in only human and mouse; HR = conserved expression only in HR; H = developmental expression not conserved between humans and either rodent species. For all conservation analyses, only orthologs that mapped one-to-one from rodent to human were included in the analysis. * indicates significant difference (*P* < 0.05) for Candidate genes in a given dataset compared to all other genes not in any dataset, either with Mann–Whitney *U* test (sequence similarity, dN/dS) or Fisher's exact test (developmental expression). Select outliers are labeled. Extreme outliers from “Other” genes have been removed from the dN/dS plots for clarity: *CDR1* (mouse dN/dS = 98.98) and *LPA* (rat dN/dS = 3.49).

We propose that therapeutically relevant Candidate genes are conserved between rodents and humans, and as such evolutionary conservation is a measure to prioritize RNA-seq candidates. For Carpenter, the Candidate genes showed higher sequence similarity to human orthologs compared to all other orthologous pairs in the genome, and this was true for both mouse-human orthologs (Mann–Whitney *U* = 513116, *n*_1_ = 536, *n*_2_ = 16078, median_1_ = 91.3, median_2_ = 86.9, *P* < 0.001) and rat-human orthologs (Mann–Whitney *U* = 4569745, *n*_1_ = 511, *n*_2_ = 15292, median_1_ = 90, median_2_ = 86.1, *P* < 0.001; Fig. 3a). However, Candidate genes from the Walker dataset showed no overall difference in either mouse-human (Mann–Whitney *U* = 764458, *n*_1_ = 99, *n*_2_ = 16078, median_1_ = 86.1, median_2_ = 86.9, *P* = 0.498) or rat-human (Mann–Whitney *U* = 707803, *n*_1_ = 95, *n*_2_ = 15292, median_1_ = 85.8, median_2_ = 86.1, *P* = 0.667) sequence similarity compared to all other orthologs (Fig. 3a, Supplementary Table 6).

As with sequence similarity, low dN/dS is a measure of conserved function. Again, the Carpenter dataset showed significantly lower dN/dS values for both mouse (Mann–Whitney *U* = 2973300, *n*_1_ = 511, *n*_2_ = 14498, median_1_ = 0.0730, median_2_ = 0.111, *P* < 0.001) and rat (Mann–Whitney *U* = 2691170, *n*_1_ = 487, *n*_2_ = 13722, median_1_ = 0.0763, median_2_ = 0.111, *P* < 0.001). Candidate orthologs (Fig. 3b) compared to all other orthologous pairs. This suggests that most of the Candidate genes from the Carpenter dataset are under purifying selection, i.e. under evolutionary pressure to maintain consistent coding sequences across the tested species. Meanwhile, the Walker dataset showed no significant difference in dN/dS values for Candidate genes compared to other orthologs for either mouse (Mann–Whitney *U* = 633448, *n*_1_ = 94, *n*_2_ = 14498, median_1_ = 0.0987, median_2_ = 0.111, *P* = 0.239) or rat (Mann–Whitney *U* = 531804, *n*_1_ = 87, *n*_2_ = 13722, median_1_ = 0.0961, median_2_ = 0.111, *P* = 0.237).

We investigated conserved developmental trajectory as another measure of conservation for prioritizing DEGs from RNA-seq data, as genes that have similar expression patterns across development may be more likely to be functionally conserved across species in this context. The statistical analysis of all genes excluded those that had no data available or that had data only for mouse-human or rat-human comparisons and not the other rodent species (Cardoso-Moreira *et al.* 2020), though individual conservation results for each gene are presented in Supplementary Table 6. Fisher's exact tests indicated no difference in the developmental trajectory conservation of the Candidate genes in the forebrain compared to all other genes from the Carpenter (*n_1_* = 330, *n_2_* = 6442, *P* = 0.144) and Walker (*n_1_* = 47, *n_2_* = 6442, *P* = 0.8931) (Fig. 3c). However, the majority of Candidate genes with complete available data showed conservation in their developmental expression in the forebrain of human vs mouse and rat (83.9% for Carpenter and 80.9% for Walker). For those genes that had available data for both HM, with or without accompanying rat data, this proportion was even higher, with 349/395 (88.4%) Carpenter genes and 40/48 (83.3%) of Walker genes having shared developmental expression trajectories between HM (Supplementary Table 6).

For each Conservation measurement (sequence similarity, dN/dS, developmental conservation), genes were categorized as having high, medium, or low conservation (Table 2, Supplementary Table 6). For sequence similarity, genes were categorized as high, medium, and low if they had similarity greater than or equal to 90%, less than 90%, and greater than or equal to 80%, or less than 80%, respectively. For dN/dS scores, genes were considered to have High conservation if they had values below the median of Candidate genes in each dataset, Medium conservation at any other nonoutlier value, and low if they were outliers (<quartile 1 (Q1)—1.5 × interquartile range (IQR) or >Q3 + 1.5 × IQR). dN/dS values were categorized as “High” if they were missing due to 100% sequence similarity, and not categorized in any other cases of missing data. Developmental conservation was categorized as High if the gene shared its developmental expression pattern in HMR, Medium if its temporal expression in humans was shared with either mouse (HM) or rat (HR), and Low for no conservation with either rodent species (H). Genes with no data for both the mouse–human and rat–human comparisons were not categorized. For genes where data was available for one rodent species and not the other, they were categorized as Medium for shared developmental expression in the forebrain with that species, and Low if they did not.

**Table 2.** Prioritization of a select subset of “Candidate” genes from the Carpenter and Walker datasets.

Dataset	Human Gene symbol	Conservation prioritization					Expression prioritization		
		Mouse-human sequence similarity	dN/dS with mouse	Rat-human sequence similarity	dN/dS with rat	Developmental conservation in forebrain	Brain specificity score	Brain region enrichment	Mean CNS expression (TPM)
Both	*CARTPT*	High	High	High	High	High	High	High	High
Carpenter	*BCAS1*	Low	Low	Low	Low	High	Medium	Medium	High
Carpenter	*GABRG2*	High	High	High	High	High	Medium	Medium	Low
Carpenter	*GAD2*	High	High	High	High	High	High	High	High
Carpenter	*KCTD17*	Low	Medium	Low	High	High	High	High	High
Carpenter	*KIF5A*	High	High	High	High	High	High	High	High
Carpenter	*KLHDC2*	High	High	High	High	Low	Low	Low	Medium
Carpenter	*LIN7B*	High	High	High	High	High	Medium	Medium	High
Carpenter	*LYPD1*	High	High	High	High	N/A	High	High	Medium
Carpenter	*MOBP*	Low	Medium	Low	Medium	High	Medium	Medium	High
Carpenter	*MUC3A*	Low	Medium	Low	N/A	Low*^a^*	Medium	Medium	Low
Carpenter	*RRAS*	High	High	High	High	Medium	Medium	Medium	High
Carpenter	*XAF1*	Low	Low	Low	Low	Medium*^a^*	Medium	Medium	Low
Carpenter	*ZDBF2*	Low	Low	Low	Low	N/A	Medium	Medium	Low
Walker	*BST2*	Low	High	Low	High	N/A	Medium	Medium	High
Walker	*CFAP74*	Low	Low	Low	Medium	High	Low	Low	Low
Walker	*DHX33*	High	High	High	High	High	Low	Low	Low
Walker	*DPYSL2*	Medium	High	High	High	High	Medium	Medium	High
Walker	*GADD45G*	High	High	High	High	Low	Medium	Medium	High
Walker	*GPR101*	Low	Medium	Low	Medium	N/A	High	High	Low
Walker	*HSPA5*	High	High	High	High	High	Medium	Medium	High
Walker	*PPFIA4*	High	High	High	High	High	Medium	Medium	High
Walker	*PPP1R15A*	Low	Low	Low	Low	Low	Medium	Medium	High
Walker	*PRKCG*	High	High	High	High	N/A	High	High	High
Walker	*SOD3*	Low	High	Low	High	High	Medium	Medium	Medium
Walker	*SPRED3*	High	High	Medium	Medium	N/A	High	High	Low

Genes were categorized as High, Medium, and Low priority for each conservation and brain expression metric. For clarity, numerical values have been removed from the table to emphasize the prioritization category; see Supplementary Table 6 for the numerical values as well as prioritization for all genes not shown in this table. Sequence similarity was measured as the percentage match between a rodent gene and its human ortholog. dN/dS values indicate the ratio of nonsynonymous (dN) to synonymous (dS) substitutions when comparing the rodent gene to its human ortholog. Complete data are listed here, even for orthologs that do not map one-to-one and were not included in the statistical analyses. Data from Cardoso-Moreira *et al*. (2020) was used to determine conservation of gene expression patterns across development in the forebrain. HMR = conserved expression across development in HMR; HM = conserved expression in only human and mouse; HR = conserved expression only in HR; H = developmental expression not conserved between humans and either rodent species. Developmental conservation assignments listed in this table are based on this available data only, assuming developmental expression is not conserved with the untested species. Brain specificity was calculated as the log_2_ FC of the total expression in CNS tissues compared to non-CNS tissues, using data from the GTEx database. The brain region(s) with the highest expression was determined based on post hoc Tukey's tests (Supplementary Table 9). See Supplementary File S1 for brain region abbreviations. Mean brain expression was calculated by obtaining the mean expression in TPM for each brain tissue across all individuals regardless of age, then calculating the grand mean.

Indicates genes that do not have complete developmental expression data available, i.e. genes that only had data comparing HM (*MUC3A*) or HR (*XAF1*).

Though the Candidate genes overall are not more or less evolutionarily conserved than other genes, there are individual genes that would make poor candidates based on their conservation. For example, some Candidate genes had very low sequence similarity and/or high dN/dS values, including *BCAS1*, *MUC3A*, *XAF1*, and *ZDBF2* (Carpenter) along with *BST2*, *CFAP74*, *PP1R15A*, and *SOD3* (Walker), among others (Fig. 3, Table 2, Supplementary Table 6). However, some of these genes are conserved in their developmental expression in the forebrain both between HM and HR (e.g. *BCAS1*, *CFAP74, SOD3*). Importantly, while many genes that have high sequence similarity and low dN/dS values are developmentally conserved, there are several exceptions, such as *KLHDC2* (Carpenter) and *GADD45G* (Walker), which are not developmentally conserved with either mouse or rat but have >95% sequence similarity. This emphasizes the importance of considering functional conservation when selecting a candidate gene, as sequence similarity and dN/dS values provide only a partial picture.

### Narrowing candidate genes by brain specificity and brain tissue- and sex-specific expression

**Fig. 4.**
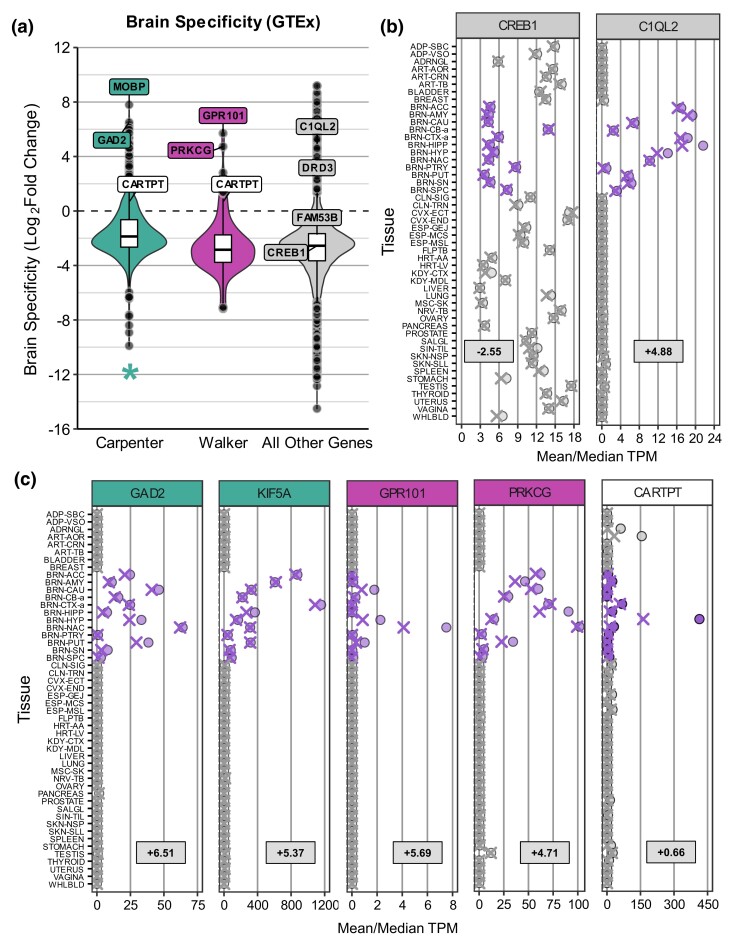
Brain specificity of candidate genes. a) Brain specificity, as measured by log2 FC of expression in CNS tissues vs all other tissues, of Candidate genes (orange) compared to all other genes (gray) in GTEx V8. Select genes are labeled, including several established genes in clinical SUDs and/or preclinical models of substance abuse—*CREB1* (Carlezon *et al.* 1998; McClung and Nestler 2003), C1QL2 (Gelernter *et al.* 2014; Huggett and Stallings 2020), *DRD3* (Neisewander *et al.* 2014; Powell *et al.* 2018; Powell, Namba, *et al.* 2020), and *FAM53B* (Gelernter *et al.* 2014). * indicates significant difference (*P* < 0.05) for Candidate genes in a given dataset compared to all other genes not in any dataset with Mann–Whitney *U* test. Mean and median expression in TPM, indicated by X's and circles, respectively, are shown for b) 2 genes implicated in SUDs in animal models or human subjects and c) 5 Candidate genes with high brain specificity, using all available samples from GTEx. *GAD2* and *KIF5A* are from the Carpenter dataset (teal), *GPR101* and *PRKCG* are from Walker (purple), and *CARPT* is shared between the 2 datasets (white). For b) and c) the overall brain specificity score for each gene is also listed. Purple and gray indicate CNS and non-CNS tissues, respectively. Full names for abbreviated genes and tissues are listed in Supplementary File 1.

Utilizing the GTEx dataset, we found that Candidate genes are expressed with higher brain specificity than other genes for the Carpenter (Mann–Whitney *U* = 20885557, *n*_1_ = 577, *n*_2_ = 54662, median_1_ = −1.87, median_2_ = −2.55, *P* < 0.001) dataset after CNS- and body-exclusive genes were removed (Fig. 4a). However, the Walker genes did not have significantly different brain specificity compared to all other genes (Mann–Whitney *U* = 2940300, *n*_1_ = 112, *n*_2_ = 54662, median_1_ = −2.84, median_2_ = −2.55, *P* = 0.47). Figure 4b depicts the overall gene expression across tissues for 2 genes previously implicated in SUDs research, *CREB1* (Carlezon *et al.* 1998; McClung and Nestler 2003) and *C1QL2* (Gelernter *et al.* 2014; Huggett and Stallings 2020). Figure 4c shows 2 select human orthologs from each dataset that have a positive brain specificity score and enriched expression in reward regions (*GAD2*, *KIF5A*, *GPR101*, *PRKCG*), as well as one Candidate gene that was shared between datasets that meet the same criteria (*CARTPT*). While most of these genes have a mean CNS expression > 10 TPM, GPR101 is expressed at lower levels (mean = 1.10 TPM and <10 TPM in the region with highest expression) and may therefore be a poorer Candidate gene than the others.

Next, we probed for possible tissue differences in brain expression of Candidate genes, as genes with brain-specific gene expression are predicted to have fewer off-target affects in therapies, as well as potential sex differences. Using age-matched data for males and females ≥55 years, we performed 2-way ANOVAs by Sex and Tissue for the 12 CNS tissues. ANOVA and post hoc Tukey statistics are reported in Supplementary Tables 7–12. Most Candidate genes showed a significant effect on Tissue, with the exception of 4 Carpenter genes and 1 Walker gene. Post hoc Tukey's tests were used to determine which brain tissue(s) showed enriched expression of a gene relative to others and to generate compact letter displays (CLDs) to cluster tissues with similar expression levels (e.g. “a” has the highest expression, “b” has the second-highest expression, and so on). For example, *KIF5A* shows higher expression in the cortex relative to all other tissues and is denoted with only “a”; however, for *DPYSL2*, the spinal cord has the highest expression (“a”) compared to all other CNS tissues except the cerebellum (“ab”). We then categorized genes as having “enriched” expression in specific brain tissue(s) if the CLD included the letter “a”.

There were 179 genes that had a significant main effect on Sex (Carpenter: 158/577; Walker: 28/112), including some that overlapped between datasets. Of these, the majority (140; 78.2%) showed higher expression in males than females, likely because the Candidate genes were obtained from datasets using male rodents. There were also 55 genes across the datasets that genes also showed a significant Sex:Tissue interaction. For example, *CARTPT*, which is shared and regulated in the same direction for Carpenter and Walker, has higher expression in males in the caudate.

The Candidate genes were categorized as High, Medium, or Low priority based on the brain expression data. For brain specificity and region enrichment, the following criteria were used for priority designation: High—brain specificity >0 and the enriched expression in reward regions, but not cerebellum or spinal cord, according to post hoc Tukey's tests. Medium—brain specificity >0 but enriched expression only in nonreward reward regions or not enriched in any specific regions, or brain specificity <0 but enriched expression in reward regions; Low—brain specificity ≤ 0 and has enriched expression only in cerebellum and/or spinal cord. The following tissues were considered reward-related: anterior cingulate cortex, amygdala, caudate, frontal cortex, hippocampus, hypothalamus, NAc, pituitary gland, putamen, and substantia nigra. The grand mean of expression (TPM) for all brain tissues was also calculated for each gene, and genes were categorized as High, Medium, and Low for this metric if their expression was ≥ 20, ≥ 10, and <20, or <10 TPM, respectively.

Using this approach, we found genes that, if they had been identified with higher confidence in each dataset (i.e. with correction for multiple testing using FDR), would make exciting candidates to explore for future study (Table 2, Supplementary Table 6). Though most of these genes do not show sex differences in brain expression for healthy human adults, for those that do (e.g. *CARTPT*), extra consideration should be made to design any preclinical experiments with adequate power to detect possible sex differences. In addition, some Candidate genes have higher expression in the brain than the rest of the body (107 Carpenter and 11 Walker genes), including *MOBP*, *KIF5A*, *LYPD1*, and *GAD2* (Carpenter), *GPR101*, and *PRKCG* (Walker) among others. However, post hoc Tukey's tests indicate some genes with higher expression in the brain, including *BCAS1* (Carpenter), *DPYSL2*, and *PPFIA4* (Walker) are preferentially expressed in the spinal cord or cerebellum, which are CNS regions involved in motor control that are not conventionally associated with SUDs. However, other genes are highly expressed in reward-related regions, such as Carpenter genes *GAD2* and *LYPD1* (both preferentially expressed in NAc) as well as *KIF5A* (preferentially expressed in the cortex). Similarly, Walker genes *GPR101* and *PRKCG* are both preferentially expressed in NAc, and *SPRED3* is most expressed in the pituitary. *CARTPT*, a common Candidate gene between the datasets, is most highly expressed in the hypothalamus. Many other Candidate genes do not have high brain specificity, but within the brain are most expressed in reward-related regions. This includes Carpenter gene *LIN7B* (highest expression in pituitary gland, hypothalamus, and NAcNAc) and Walker gene *HSPA5* (highest in pituitary gland).

### RNA-seq power analysis

**Fig. 5.**
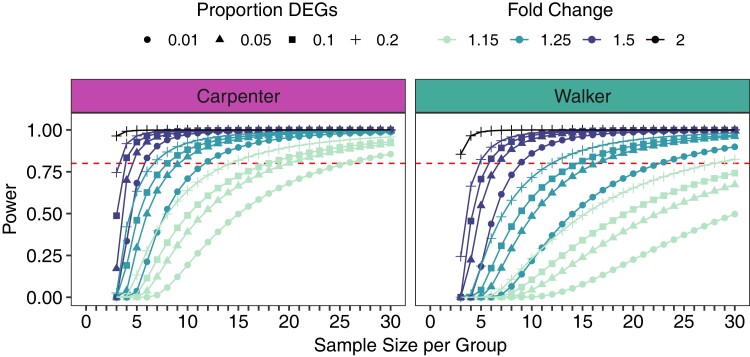
Estimated necessary sample size per group using the r package *ssizeRNA*. The number of samples, up to 30, that is necessary to reach varying levels of power are shown for the Carpenter (teal) and Walker (purple) datasets. Dashed red lines indicate 0.8 power with FDR < 0.05. Power varies with the proportion of DEGs and their FC (FC). Because these metrics were not directly attainable for these datasets, varying proportions of DEGs and fold changes were tested and indicated with different shapes and colors, respectively. For these theoretical calculations, FC is relative to all DEGs (e.g. if the proportion of DEGs is 0.2 and FC is 2, it is expected that 20% of genes are DEGs, all with FC of at least 2). For clarity, the sample size estimations for FC of 2 are shown only for a proportion of DEGs equaling 0.2, though similar sample sizes are required for 0.05 or 0.1. Vertical gray bars indicate the actual sample size per group for each study after removing outliers.

Because almost no DEGs were identified for each study using an FDR cutoff, we calculated the sample size per group required to reach 0.8 power for each dataset with FDR <0.1. Estimated sample size varied for each dataset based on the expected proportion of DEGs and their FC values (Fig. 5). For example, assuming a small effect size for each DEG (FC of 1.25) but a high proportion of DEGs (0.2, or 20%), the datasets were estimated to need 7 (Carpenter) and 12–13 (Walker) samples per group. Calculating for both a large effect size (FC of 2) and high proportion of DEGs (0.2, or 20%), the datasets were each estimated to need only about 3–4 samples per group. In a more realistic scenario, where fewer genes are differentially expressed (5%, corresponding to 500 genes for every 10,000), each with a small FC difference (1.25), the estimated necessary sample size varies more between datasets, with about 8–9 for Carpenter, but 16–17 for Walker. This may be due to higher variation in gene expression between samples for the Walker dataset than Carpenter, as Walker shows higher mean dispersion across all genes (0.028) than Carpenter (0.015) (Supplementary Table 2). Notably, with the differential expression analysis performed here, DEGs that met the uncorrected *P-*value threshold (*P* < 0.05) also had a low overall mean FC (Carpenter: 1.17; Walker: 1.39).

### Pipeline demonstration on a 1d cocaine abstinence dataset

Lastly, we tested the pipeline on the Engeln dataset (Engeln *et al.* 2022), in which VP tissue was collected from male mice 24 hr after cocaine or saline SA (*n* = 4/group). The authors found 363 DEGs between S1 and C1 mice. Of these, 314 had rat orthologs corresponding to 309 rat genes, and 316 had human orthologs corresponding to 310 human genes. Supplementary Table 13 contains the full prioritization results, including High, Medium, and Low priority labels, for each of the 310 human orthologs from the Engeln dataset. Supplementary Tables 7–12 show the complete ANOVA and Tukey's test results for the GTEx comparisons.

For conservation prioritization, these genes showed higher sequence similarity than all other genes both when comparing mouse to human (Mann–Whitney *U* = 2878718, *n*_1_ = 296, *n*_2_ = 16408, median_1_ = 90.4, median_2_ = 87.0, *P* < 0.001) and rat to human (Mann–Whitney *U* = 2641146, *n*_1_ = 283, *n*_2_ = 15599, median_1_ = 89.8, median_2_ = 86.2, *P* < 0.001), as well as significantly lower dN/dS values for both mouse (Mann–Whitney *U* = 1607880, *n*_1_ = 269, *n*_2_ = 14826, median_1_ = 0.078, median_2_ = 0.110, *P* < 0.001) and rat comparisons (Mann–Whitney *U* = 1433077, *n*_1_ = 251, *n*_2_ = 14037, median_1_ = 0.0791, median_2_ = 0.110, *P* < 0.001; Supplementary Figure 4a, b). Similarly, the genes showed higher developmental conservation in the forebrain than other genes (*n_1_* = 152, *n_2_* = 6659, *P* = 0.0315), with only 7 genes that were not conserved in their developmental trajectory between humans and either rodent species (Supplementary Figure 4c).

For the brain expression component of the pipeline, human orthologs of Engeln DEGs showed significantly higher brain specificity than all other genes in the GTEx dataset (Mann–Whitney *U* = 5002067, *n*_1_ = 307, *n*_2_ = 23872, median_1_ = 90.0, median_2_ = 81.0, *P* < 0.001; Supplementary Figure 4d). This included several genes known to be important for brain function, such as the glial marker *GFAP*, vasopressin (encoded by *AVP*). In addition, one of the DEGs from the Engeln dataset was *C1QL2*, which has been identified as a cocaine dependance risk gene through a human genome-wide association study (Gelernter *et al.* 2014). Of the 305 Engeln genes that were present in GTEx, 303 had a significant main effect on tissue, 58 had a significant main effect on Sex, and 42 had a significant Sex:Tissue interaction. Of those that had a Sex effect, only 5 were higher in females than males.

### Comparison between datasets

**Fig. 6.**
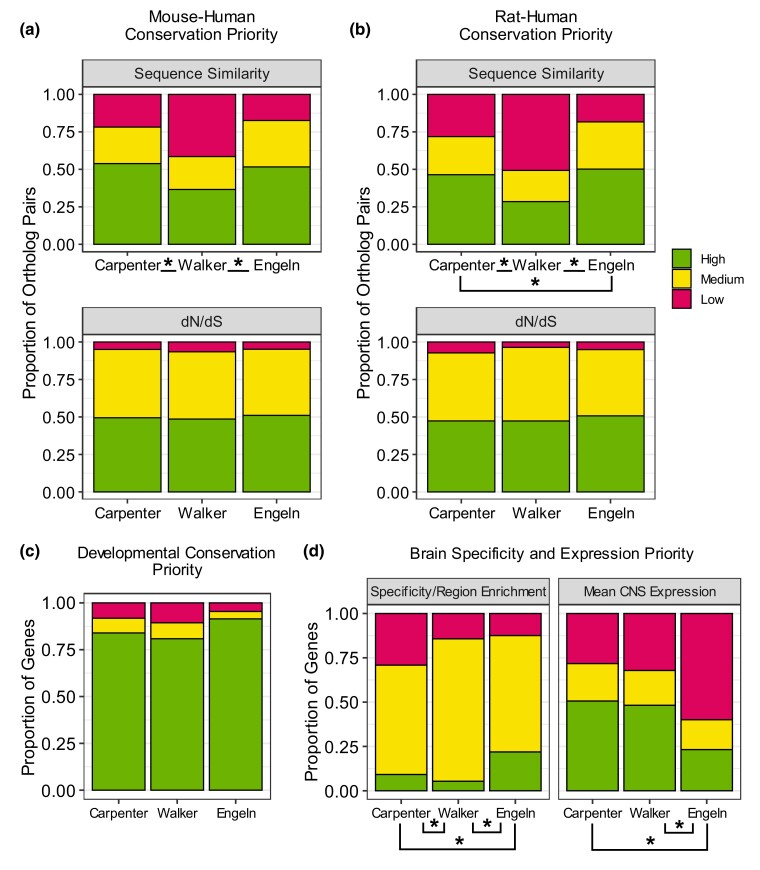
Distribution of priority scores across datasets. The distributions of High (green), Medium (yellow), and Low (red) priority scores for each pipeline metric were compared for Candidate genes or ortholog pairs from the Carpenter, Walker, and Engeln datasets. * indicates significant difference in priority score distribution (*P* < 0.05) between 2 datasets using Fisher's exact test after a significant Fisher's exact test considering all 3 datasets together.

The distribution of Low, Medium, and High priority scores was compared between the Carpenter, Walker, and Engeln datasets (Fig. 6). For both mouse–human and rat–human sequence similarity, there was a significantly different priority score distribution between datasets (both *P* < 0.001; Fig. 6a, b). Follow-up Fisher's exact tests showed that Carpenter and Walker were each significantly different from each other for both the mouse and rat sequence similarity metrics, as were Engeln and Walker (both *P* < 0.001). Comparing Carpenter and Engeln, there was no difference between priority distributions for mouse-human sequence similarity (*P* = 0.068) though there was a significant difference for rat (*P* < 0.01). Overall, the Carpenter and Engeln datasets had more orthologous gene pairs that were High or Medium priority than Walker. There was no significant difference between datasets for mouse dN/dS (*P* = 0.936), rat dN/dS (*P* = 0.488), or developmental conservation (*P* = 0.165; Fig. 6a-c).

We also compared the priority score distribution for 2 brain expression metrics: a combined brain specificity and region enrichment score, and mean CNS expression. For brain specificity and enrichment, the 3 datasets had different distributions in priority scores (*P* < 0.001). Subsequent Fisher's exact tests showed significantly different distributions between Carpenter and Walker Carpenter and Engeln, and Walker and Engeln (all *P* < 0.001). Looking across datasets, Engeln had more High priority and fewer Low priority genes than Carpenter and Walker. For mean CNS expression, there was also a significant difference between the 3 datasets (*P* < 0.001), with follow-up tests showing significant differences between Carpenter and Engeln as well as Walker and Engeln (both *P* < 0.001), but not Carpenter and Walker (*P* = 0.7122). Interestingly, the Engeln dataset had more Low priority and fewer High and Medium priority genes for mean CNS expression than the other 2 datasets.

## Discussion

We analyzed 2 RNA-seq datasets from rodents that underwent cocaine SA and prolonged forced ABS, with the goal to prioritize DEGs from each study related to ABS-induced changes in drug motivation and with high translational potential. We suggest a next step toward further refining lists of DEGs is prioritizing those with evolutionary and functional conservation and preferential expression in brain tissues. Using human orthologs of the DEGs (*P* < 0.05) as “Candidate” genes, we developed a novel pipeline to categorize these genes as having High, Medium, or Low priority to determine which would make better candidates for translational research and validation (Table 2, Supplementary Table 6). In this discussion, we focus on a subset of the Candidate genes from each dataset to demonstrate utility of the pipeline (Table 2). However, because the ability to detect positive DEGs is limited by the low sample sizes and power in each dataset, we caution that these Candidate genes should be taken as demonstration of the prioritization pipeline and not specific genes of interest, and discuss other necessary improvements to RNA-seq analysis that will bolster the use of this technology in preclinical research.

Our primary goal was to develop and demonstrate a pipeline for improving the prioritization of DEGs in preclinical research. In each of our test datasets, we moved forward with a list of DEGs as “Candidate” genes from each study to illustrate the utility of pruning these lists based on several metrics. Using this novel pipeline, we found that several of our test genes are both highly conserved and are enriched in brain tissues involved in reward (e.g. *CARTPT, GAD2*, *GADD45G*, *KIF5A*, and *LYPD1*) (Table 2). Several other Candidate genes are well-conserved and show high brain specificity, though they have enriched expression in nonreward regions (e.g. *BCAS1*, *GABRG2*, *MOBP*, *PPFIA4*). This prioritization strategy also identified several genes that would make poorer therapeutic targets because they are less conserved (e.g. *GPR101*, *MUC3A*, *PPP1R15A*), are not brain-specific and have elevated expression in nonreward tissues (e.g. *DHX33*, *KLHDC2*) or all 3 (e.g. *CFAP74*).

To narrow down genes by translational value, we reasoned that therapeutic targets should have shared evolutionary conservation between mouse, rat, and human. We found that overall, the Candidate genes had generally high sequence similarity and low dN/dS values (i.e. high conservation) between their rodent and human orthologs (Figs. 3a and b), and for the Carpenter dataset, these DEGs were significantly more conserved in these metrics than all other genes. Generally, it appears that these genes are evolving under strong purifying selection and that there are evolutionary pressures to retain the same amino acid encoding even when the exact sequences diverge. While all of our Candidate genes generally had low or moderate dN/dS values (<0.8 for both mouse and rat), there are several outliers with higher dN/dS values that were categorized as Low priority for their datasets including *PPP1R15A*, *XAF1*, and *ZDBF2* (Fig. 3a, Table 2). Though these also have Low sequence similarity (< 45% for both mouse–human and rat–human orthologs), several other genes (e.g. *BST2*, *GPR101*, *KCTD17, MOBP, SOD3*) show Low (< 80%) or sequence similarity but still have low or moderate dN/dS values (Low or Medium priority for their datasets). Thus, despite some changes in their sequences, the genes are still likely to be under evolutionary constraint that maintains much of their protein conformation and perhaps their molecular function as well. By considering both sequence similarity and dN/dS values, we can discard genes as poor candidates due to low sequence similarity while tentatively retaining genes with low sequence similarity but low dN/dS values.

Similarly, our analysis revealed that most of the Candidate genes have conserved expression across matched developmental stages in the forebrain of HMR (Fig. 3c), though overall they are not conserved more than other genes. However, by adding this with dN/dS we can identify genes with relatively high sequence similarity (High or Medium priority) and low dN/dS (High priority), but which show divergent developmental expression (e.g. *GADD45G* and *KLHDC2*), suggesting their function may not be conserved across species, at least in the context of development. Given that many known addiction-related genes are strongly implicated in brain development, and abnormal neurodevelopment can increase risk of SUDs in adulthood (McCrory and Mayes 2015), genes that are not conserved across development seem less likely to have translational utility. Of note, some Candidate genes show conserved developmental expression patterns between humans and either human or mouse, but not both, such as *RRAS* (Carpenter). Genes such as these may be well-suited for study in mouse models, but less so in rats. Genes that are highly conserved across all metrics, such as *CARTPT*, *DHX33*, *GABRG2*, *GAD2*, *HSPA5*, *KIF5A*, *LIN7B*, and *PPFIA4*, may better candidates for research across preclinical rodent models (Table 2).

We further propose to narrow translational rodent targets based on whether their orthologs in humans have enriched expression across human brain tissues. Within the brain, genes that are most highly expressed in reward regions such as the amygdala, NAc, and the dorsal striatum are appealing candidates for future study (e.g. *KCTD17*, *KIF5A*, *LIN7B*, and *PRKCG* show elevated expression in some or all of these regions; Table 2). However, these genes do not have higher brain specificity than other genes, and thus we have categorized them as Medium priority based on brain expression (Fig. 4a, Table 2). While a gene does not need to be brain-specific to have an impact on reward processing and behavior (e.g. the well-studied addiction gene *CREB1* is not brain-specific, Fig. 4b), ubiquitous expression may make these genes difficult to target with therapeutics without negative off-target effects in other tissues. However, several Candidate genes do show elevated expression in the brain compared to non-CNS tissues. For example, we find the myelin oligodendrocyte gene *MOBP* (Carpenter) has 8-fold higher expression in the brain relative to all other tissues, suggesting targeting its expression may have a stronger effect in the brain than the rest of the body. In addition, *MOBP* has already been implicated in cocaine abuse (Albertson *et al.* 2004, 2006). However, *MOBP* is most highly expressed in spinal cord tissue, and therapeutics that target this gene may provoke cognitive and sensory/motor side effects, which is another important consideration in selecting genes for further study. Other Candidate genes score as High for brain expression prioritization because they show brain-specificity and are more highly expressed in reward regions, and have a mean CNS expression above 20 TPM (e.g. *CARTPT*, *GAD2*, *KCTD17*, *KIF5A*, *LYPD1*, *PRKCG*). Insights from prior literature help further prioritize these Candidate genes. For example, *KCTD17* (Carpenter) and other *KCTD* family members regulate cyclic adenosine monophosphate signaling (Muntean *et al.* 2022) and are implicated in neurological disorders (Teng *et al.* 2019). As well, *CARTPT* (Carpenter, Walker) is a prepropeptide related to the addiction gene *CART* (Cocaine and Amphetamine Regulated Transcript) (Kuhar *et al.* 2005; Ong and McNally 2020). These Candidate genes are generally well conserved, though *KCTD17* has low sequence similarity for both mouse and rat (Low priority for these metrics). Genes that are not brain-specific and show enriched expressed in nonreward regions of the brain (e.g. cerebellum and/or spinal cord) are Low priority Candidate genes (e.g. *CFAP74, DHX33, KLHDC2*). Still, when selecting candidate genes for preclinical rodent research, conservation is of greater importance than brain expression. Some of these genes are already Low priority for one or more conservation metrics, while others such as *DHX33* are highly conserved. Thus, by combining evolutionary conservation and brain expression data, researchers can select the most translationally relevant genes from their RNA-seq datasets.

In addition to improving candidate gene prioritization, we advocate for additional improvements to RNA-seq analysis and reporting in preclinical research. Our analysis suggests that both test datasets were underpowered to detect true DEGs after correcting for multiple testing. However, this issue is not limited to these data but instead reflects a greater issue in neuroscience RNA-seq at large. Differential expression, pathway, and gene set enrichment analyses based on RNA-seq data are regularly published with low sample sizes (<10 per group) and uncorrected *P-*values (for example, see our own RNA-seq study in rats (Powell, Vannan, *et al.* 2020)). This could be due to a lack of funds to support larger RNA-seq analyses, or because researchers may feel indirect pressure to avoid correcting for multiple testing appropriately because doing so would severely reduce or entirely eliminate any DEGs, as we show here (for another example, see Mukamel 2021). Importantly, the use of uncorrected *P-*value thresholds for RNA-seq analysis, which tests across thousands of genes, is likely to produce many false positive DEGs. With this statistical threshold (*P* < 0.05), we identified 6.3% (633/10,026) and 1.4% (139/9,948) of the tested genes in each dataset as DEGs for Carpenter and Walker, respectively. However, many or most of these genes may be false positives, as with *P* < 0.05 we would expect about 500 false positive DEGs for every 10,000 genes tested (5%). Researchers may try to mitigate the issue of false positive DEGs gleaned from inappropriate statistical testing by spot-checking the validity of a small subset of DEGs by comparison to prior literature or with RT-qPCR. Perhaps the best approach to confirming RNA-seq results is through functional validation, such as direct manipulation of a target gene through pharmacological or viral interventions. For example, the Carpenter and Engeln studies used their results to inform follow-up experiments validating an individual DEG, *Nr4a1* (Carpenter *et al.* 2020; Engeln *et al.* 2022). Still, confirming RNA-seq results for a single gene or a small set of genes does not automatically indicate that all identified DEGs are true positives, and it is not always clear whether authors report DEGs that failed to validate. Another issue is that the use of uncorrected *P-*values may result in low overlap even between highly similar datasets, as noted by other researchers (Noble 2009; Mukamel 2021), reducing the field's ability to compare across RNA-seq studies. This closes off a potential avenue for study, and prevents researchers from accurately comparing across datasets to find replicable changes in gene expression between similar experimental models. Moving forward in the field, researchers must focus on robust data collection and analysis in RNA-seq to improve the translational value of these datasets.

Because SUDs and other complex behaviors and diseases are likely driven by complex interactions of many genes with low effect sizes (Geschwind and Flint 2015; Prom-Wormley *et al.* 2017), it is difficult to detect reliable and meaningful transcriptome-wide changes without adequately large sample sizes. Generally, the necessary sample size for an RNA-seq study is likely to be at least the number needed for RT-qPCR validation or behavioral testing, if not more, due to the many variables in sequencing that cannot be accounted for directly and differences in study goals and design. For example, we also sought to compare our prioritization pipeline results with an FDR-corrected set of genes, so we utilized a list of DEGs (FDR < 0.05) obtained directly from a mouse cocaine SA study by Engeln *et al.* (2022). This study tested differences in VP tissue from male mice sacrificed after 24 hr ABS from saline or cocaine SA and was selected for comparison due to a general paucity of RNA-seq datasets using prolonged ABS models. With the Engeln dataset, we found many potential genes of interest with High or Medium conservation and brain specificity, including genes already implicated in SUDs such as *C1QL2* (Marees *et al.* 2020; Engeln *et al.* 2022), *AVP* (encodes vasopressin) (Godino and Renard 2018), *HTR1B* (encodes the serotonin 1B receptor) (Rocha *et al.* 1998; Neisewander *et al.* 2014; Der-Ghazarian *et al.* 2019), and the synaptic scaffolding gene *HOMER1* (Ghasemzadeh *et al.* 2003; Szumlinski *et al.* 2008; Dell’Orco *et al.* 2021). Overall, the Engeln DEGs showed significantly higher conservation and brain specificity compared to other genes (Supplementary Fig. 4). We also compared the distribution of priority scores between the Engeln, Carpenter, and Walker genes, and found that the Engeln dataset was on par with or contained more High and Medium priority genes than the other datasets across almost all metrics except mean CNS expression (Fig. 6). This could reflect genuine differences in the types of genes identified using appropriate multiple testing correction, or may instead be related to the studies’ designs, including brain regions selected and a difference between prolonged vs short ABS. With only a 24 hr ABS period, it is possible that the direct pharmacological effects of cocaine will manifest in widespread changes in the brain that are consistent across subjects, such that the effect sizes are large enough to detect even at low sample sizes (*n* = 4 per group in the Engeln study). For more complex behavioral models, a small sample size is less likely to be sufficient. For example, testing animals with a longer period of drug ABS is translationally relevant, but may result in smaller overall changes or large interindividual variation in gene expression that requires a higher sample size to adequately probe.

The prioritization pipeline described here serves as a companion to other work in genetic bioinformatics. For example, there are several gene databases and search tools for SUDs that curate supporting literature across species (Li *et al.* 2008; Shi *et al.* 2021; Gunturkun *et al.* 2022), though these are naturally limited to genes that have already been implicated in other research. There are also several bioinformatics studies that utilize human and rodent data in-tandem, either by leveraging publicly available datasets from multiple species (Huggett *et al.* 2020; Huggett and Stallings 2020) or by designing experiments that complement one another directly (Enoch *et al.* 2012). However, more cross-species RNA-seq studies still are needed, as noted in a 2019 white paper from the National Institute on Drug Abuse (NIDA) (Cates *et al.* 2019). Importantly, while all of these approaches provide crucial insight into species differences, functional validation of candidate genes is still a fundamental step in translating preclinical research. As such, our pipeline aims not to replace this step but to complement the validation process by narrowing down genes of interest before designing follow-up experiments in both rodents and humans.

At its best, RNA-seq allows for unbiased candidate gene discovery across the transcriptome, but the standard common practice fails to capitalize on its potential. Though it may be tempting to reduce sequencing and animal costs by lowering sample size, this is particularly problematic in RNA-seq, because gene expression trends identified with a nominal *P-*value will not necessarily hold with higher sample sizes and more robust statistics. Though financial costs have historically contributed to small sample sizes, sequencing has become less expensive in recent years, and there remains the opportunity for large-scale collaboration between labs, such as through NIH U or *P* grants (National Institutes of Health 2019). With more funding, along with better reporting of methods, results, and code by researchers, RNA-seq can become an important tool in discovery of candidate genes rather than suspect for generating unreliable DEGs (Simoneau *et al.* 2021). Further, we show here that additional steps taken after differential expression analysis, such as determining the evolutionary conservation and regional expression of candidate genes, can potentially narrow down future targets of study, with the caveat that this approach is best applied with properly powered data. Utilizing this approach, along with ensuring appropriately large sample sizes, will both bolster the reliability of individual RNA-seq studies and provide a stronger foundation for preclinical research to bridge the translational gap.

## Supplementary Material

jkad143_Supplementary_Data

## Data Availability

RNA-seq files were deposited by the original researchers in NCBI's Gene Expression Omnibus (Carpenter: GSE141520, Walker: GSE110344) and Sequencing Read Archive (Carpenter: SRP234876, Walker: SRP132477). Scripts for the proposed workflow for narrowing candidate genes are provided on GitHub (https://github.com/SexChrLab/RodentAddiction). Supplemental material available at G3 online.
