# Supplementary material for: An approach for prioritizing candidate genes from RNA-seq using preclinical cocaine self-administration datasets as a test case
Source: G3 (Bethesda). 2023 Jul 12;13(10):jkad143. doi: 10.1093/g3journal/jkad143 (PMC10542560; doi:10.1093/g3journal/jkad143)
Supplement: jkad143_Supplementary_Data [file jkad143_supplementary_data.zip › Figure_S2_-_FINAL_G3-2022-404013.pdf]

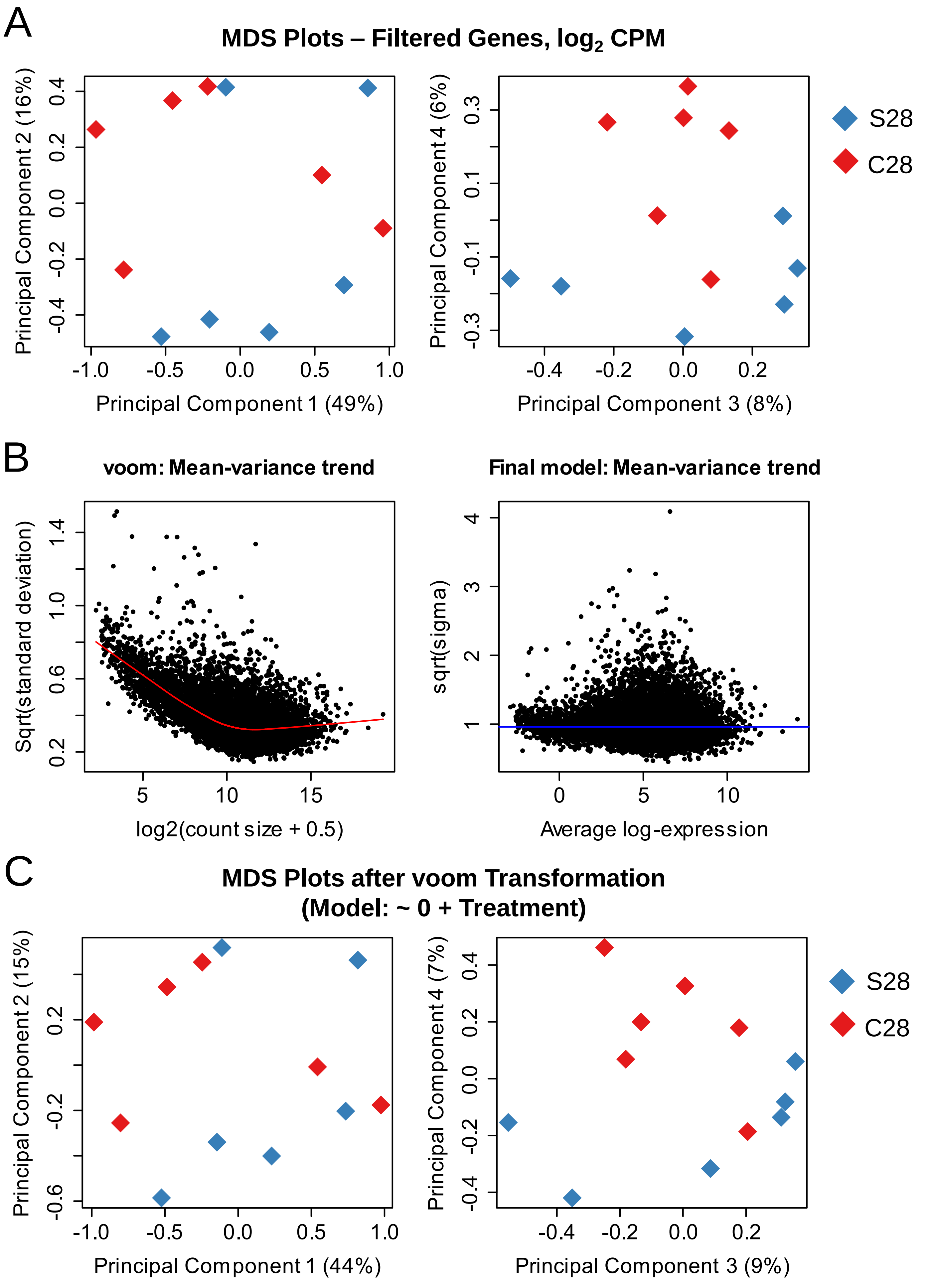

A
MDS Plots – Filtered Genes, log2 CPM
Principal Component 2 (16%)
Principal Component 1 (49%)
Principal Component 4 (6%)
Principal Component 3 (8%)
S28
C28
B
voom: Mean-variance trend
Sqrt(standard deviation)
log2(count size + 0.5)
Final model: Mean-variance trend
sqrt(sigma)
Average log-expression
C
MDS Plots after voom Transformation
(Model: ~ 0 + Treatment)
Principal Component 2 (15%)
Principal Component 1 (44%)
Principal Component 4 (7%)
Principal Component 3 (9%)
S28
C28
