# Supplementary material for: An approach for prioritizing candidate genes from RNA-seq using preclinical cocaine self-administration datasets as a test case
Source: G3 (Bethesda). 2023 Jul 12;13(10):jkad143. doi: 10.1093/g3journal/jkad143 (PMC10542560; doi:10.1093/g3journal/jkad143)
Supplement: jkad143_Supplementary_Data [file jkad143_supplementary_data.zip › File_S1_-_FINAL_G3-2022-404013.docx]

**Supplementary materials**

**Gene abbreviations**

Gene abbreviations are listed for human orthologs. Genes discussed in regards to multiple datasets may be listed twice.

*Human orthologs of discussed Candidate genes for Carpenter and Walker datasets*

BCAS1 Brain Enriched Myelin Associated Protein 1

BST2 Bone Marrow Stromal Cell Antigen 2

CARTPT Cocaine- And Amphetamine Regulated Transcript (CART) Prepropeptide

CFAP74 Cilia And Flagella Associated Protein 74

DHX33 DEAH-Box Helicase 33

DPYSL2 Dihydropyrimidinase Like 2

GABRG2 Gamma-Aminobutyric Acid (GABA) Type A Receptor Subunit Gamma 2

GAD2 Glutamate Decarboxylase 2

GADD45G Growth Arrest And DNA Damage Inducible Gamma

GPR101 G Protein-Coupled Receptor 101

HSPA5 Heat Shock Protein Family A (Hsp70) Member 5

KCTD17 Potassium Channel Tetramerization Domain Containing 17

KIF5A Kinesin Family Member 5A

KLHDC2 Kelch Domain Containing 2

LIN7B Lin-7 Homolog B, Crumbs Cell Polarity Complex Component

LYPD1 LY6/PLAUR Domain Containing 1

MOBP Myelin Associated Oligodendrocyte Basic Protein

MUC3A Mucin 3A, Cell Surface Associated

PPFIA4 Protein Tyrosine Phosphatase, Receptor Type, F Polypeptide (PTPRF), Interacting Protein (Liprin), Alpha 4

PPP1R15A Protein Phosphatase 1 Regulatory Subunit 15A

PRKCG Protein Kinase C Gamma

RRAS RAS Related

SOD3 Superoxide Dismutase 3

SPRED3 Sprouty Related EVH1 Domain Containing 3

XAF1 XIAP Associated Factor 1

ZDBF2 Zinc Finder DBF-Type Containing 2

*Human orthologs of discussed DEGs from Engeln dataset*

AVP Arginine Vasopressin

C1QL2 Complement C1q Like 2

CCDC187 Coiled-Coil Domain Containing 187

CDR1 Cerebellar Degeneration Related 1

ERVW-1 Endogenous Retrovirus Group W Member 1, Envelope

GFAP Glial Fibrillary Acidic Protein

GPR179 G Protein-Coupled Receptor 179

RESP18 Regulated Endocrine Specific Protein 18

SPINK8 Serine Peptidase Inhibitor Kazal Type 8 (Putative)

ZBDF2 Zinc Finder DBF-Type Containing 2

*Other genes*

C1QL2 Complement C1q Like 2

CART Cocaine- and Amphetamine-Related Transcript

CREB1 cAMP Responsive Element Binding Protein 1

DRD3 Dopamine Receptor D3

FAM53B Family With Sequence Similarity 53 Member B

**Tissue abbreviations**

From Gene-Tissue Expression (GTEx) V8 database. Tissues with the prefix “BRN” (brain) are located in the central nervous system (CNS).

ADP-SBC Adipose – subcutaneous SPLEEN Spleen

ADP-VSO Adipose - visceral omentum STOMACH Stomach

ADRNLGL Adrenal gland TESTIS Testis

ART-AOR Artery - aorta THYROID Thyroid

ART-CRN Artery – coronary UTERUS Uterus

ART-TB Artery – tibial VAGINA Vagina

BLADDER Bladder WHLBLD Whole blood

BRN-AMY Amygdala

BRN-ACC Anterior cingulate cortex (BA24)

BRN-CAU Caudate, basal ganglia

BRN-CB-a Cerebellar hemisphere

BRN-CB-b Cerebellum

BRN-CTX-a Frontal cortex (BA9)

BRN-CTX-b Cortex

BRN-HIPP Hippocampus

BRN-HYP Hypothalamus

BRN-NAC Nucleus accumbens, basal ganglia

BRN-PTRY Pituitary gland

BRN-PUT Putamen, basal ganglia

BRN-SN Substantia nigra

BRN-SPC Spinal cord (cervical C1)

BREAST Breast

CELL-FB Cells - cultured fibroblasts

CELL-LYM Cells - EBV-transformed lymphocytes

CVX-ECT Cervix - ectocervix

CVX-END Cervix - endocervix

CLN-SIG Colon - sigmoid

CLN-TRN Colon - transverse

ESP-GEJ Esophagus - gastroesophageal junction

ESP-MCS Esophagus mucosa

ESP-MSL Esophagus mucularis

FLPTB Fallopian tube

HRT-AA Heart - atrial appendage

HRT-LV Heart - left ventricle

KDY-CTX Kidney - cortex

KDY-MDL Kidney - medulla

LIVER Liver

LUNG Lung

SALGL Salivary gland

MSC-SK Skeletal muscle

NRV-TB Nerve - tibial

OVARY Ovary

PANCREAS Pancreas

PROSTATE Prostate

SKN-NSP Skin - not sun exposed, subrapubic

SKN-SLL Skin - sun exposed, lower leg

SIN-TIL Small intestine - terminal ileum

**Further information on discrepancies between Ensembl versions 99 and 106.** To ensure consistency across analyses, human orthologs for Carpenter, Walker, and Engeln genes were determined based on Ensembl version 106, though dN/dS values were only available for Ensembl version 99. Discrepancies in gene IDs and orthologs between versions affected a small number of genes in each dataset. Of note, this led to the categorization of the human gene gene *CDR1* (Ensembl 99 ID: ENSG00000184258; Ensembl 106 ID: ENSG00000288642), ortholog of Engeln mouse gene *Cdr1* (Mouse ID: ENSMUSG00000090546), as an “Engeln” gene for the sequence similarity analysis but part of “All Other Orthologs” for the dN/dS analysis. Because *CDR1* has an extreme value for human-mouse dN/dS (98.98; not plotted for either the Carpenter/Walker or the Engeln analyses; Figure 3, S4), we performed the dN/dS analysis for the Engeln dataset multiple ways to determine whether results for the overall set of genes were consistent irrespective of *CDR1*. Indeed, we found that dN/dS values were lower for human-mouse ortholog pairs than all other orthologs whether *CDR1* was categorized with Engeln orthologs (Mann–Whitney *U* = 1622706, *n*_1_ = 270, *n*_2_ = 14825, median_1_ = 0.0780, median_2_ = 0.110, P < 0.001), all other orthologs (Mann–Whitney *U* = 1607880, *n*_1_ = 269, *n*_2_ = 14826, median_1_ = 0.0786, median_2_ = 0.110, P < 0.001; reported in main text), or excluded from the analysis entirely (Mann–Whitney *U* = 1607880, *n*_1_ = 269, *n*_2_ = 14825, median_1_ = 0.0780, median_2_ = 0.110, P < 0.001).

**Supplementary figure legends**

**Figure S1. RNA-seq pipeline for differential expression analysis.** Both datasets were analyzed separately using the above pipeline.

**Figure S2. RNA-seq processing for Carpenter S28 vs. C28 dataset.** A, C) Multidimensional scaling (MDS) plots for the top 100 genes with the largest fold change differences between groups A) before and C) after voom transformation. Principal components 1 and 2 (left) and 3 and 4 (right) are shown, along with the percentage of variation captured by each component. Blue and red signify the S28 and C28 groups, respectively. B) limma voom was used to apply a linear model to the data. Gene expression was measured in CPM with (A, C) and without (B) log2 transformation.

**Figure S3. RNA-seq processing for Walker S30 vs. C30 dataset.** A, D) Multidimensional scaling (MDS) plots for the top 100 genes with the largest fold change differences between groups A) before and D) after voom transformation. Principal components 1 and 2 (left) and 3 and 4 (right) are shown, along with the percentage of variation captured by each component. Blue and red signify the S30 and C30 groups, respectively. Shapes represent different sequencing batches. B) limma voom was used to apply a linear model to the data, including both Treatment group and Batch, based on plots in C) which indicate the highest percentage of variance was explained by Batch. C) Variance partition plots showing the percentage of the gene expression variance explained by the variables sequencing Batch (green), sequencing Instrument (yellow), and Treatment (brown), along with Residuals (gray). Total percentages are shown on the left and are broken down by the first 10 dimensions on the right. Gene expression was measured in CPM with (A, D) and without (B, C) log2 transformation.

**Figure S4. Prioritization pipeline results for DEGs (FDR < 0.05) from the Engeln 1d ABS dataset.** A) Sequence similarity, B) dN/dS, C) developmental conservation in the forebrain, and D) brain specificity were calculated as for the human orthologs of DEGs in the Engeln dataset. Select genes are labeled. * indicates significant difference (P < 0.05) between Engeln genes compared to all other genes, either with Mann-Whitney U test (sequence similarity, dN/dS) or Fisher’s exact test (developmental expression). Select outliers are labeled, along with some genes with a previously established role in SUDs (see Figure 4). Extreme outliers from “Other” genes have been removed from the dN/dS plots for clarity: CDR1 (mouse dN/dS = 98.98) and LPA (rat dN/dS = 3.49).

**Supplementary tables and captions**

Supplementary Tables are attached in a separate Excel workbook. The first sheet of the workbook contains the table captions.
