# Supplementary figures and images for: An approach for prioritizing candidate genes from RNA-seq using preclinical cocaine self-administration datasets as a test case
Source: G3 (Bethesda). 2023 Jul 12;13(10):jkad143. doi: 10.1093/g3journal/jkad143 (PMC10542560; doi:10.1093/g3journal/jkad143)

### (1) Obtain and Trim Files

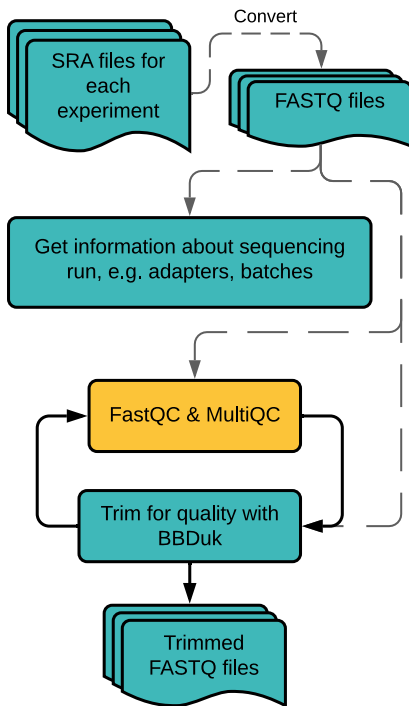

### (2) Alignment & Counting

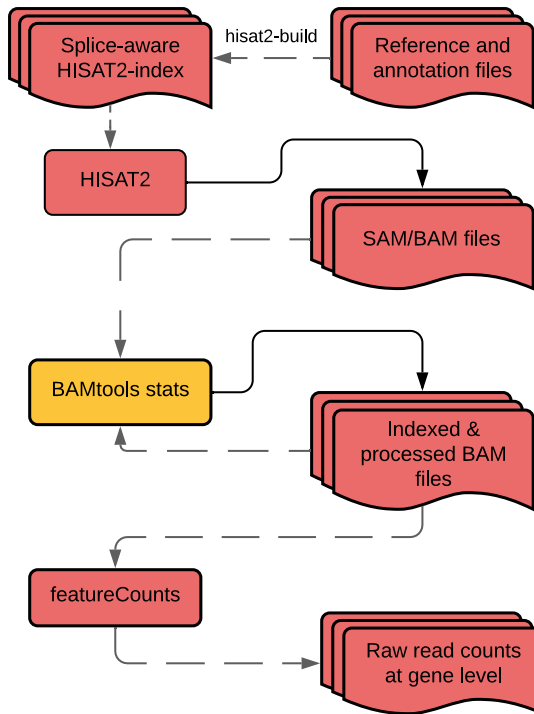

### (3) Processing and Differential Expression

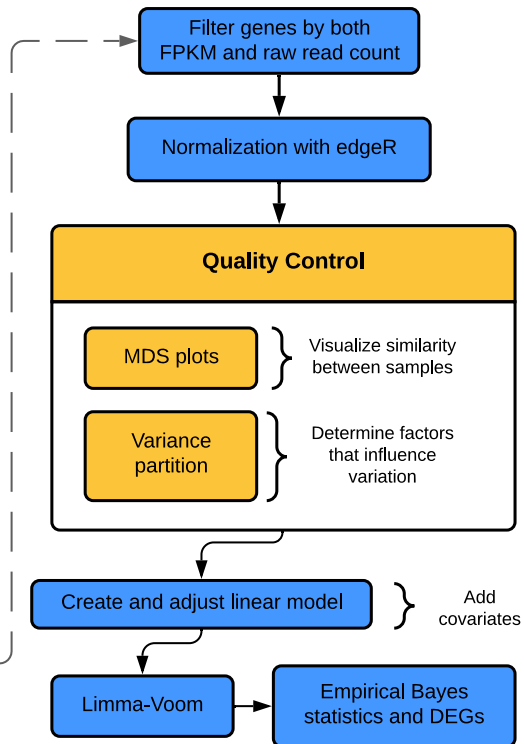

Supplement: jkad143_Supplementary_Data [file jkad143_supplementary_data.zip › Figure_S1_-_FINAL_G3-2022-404013.pdf]

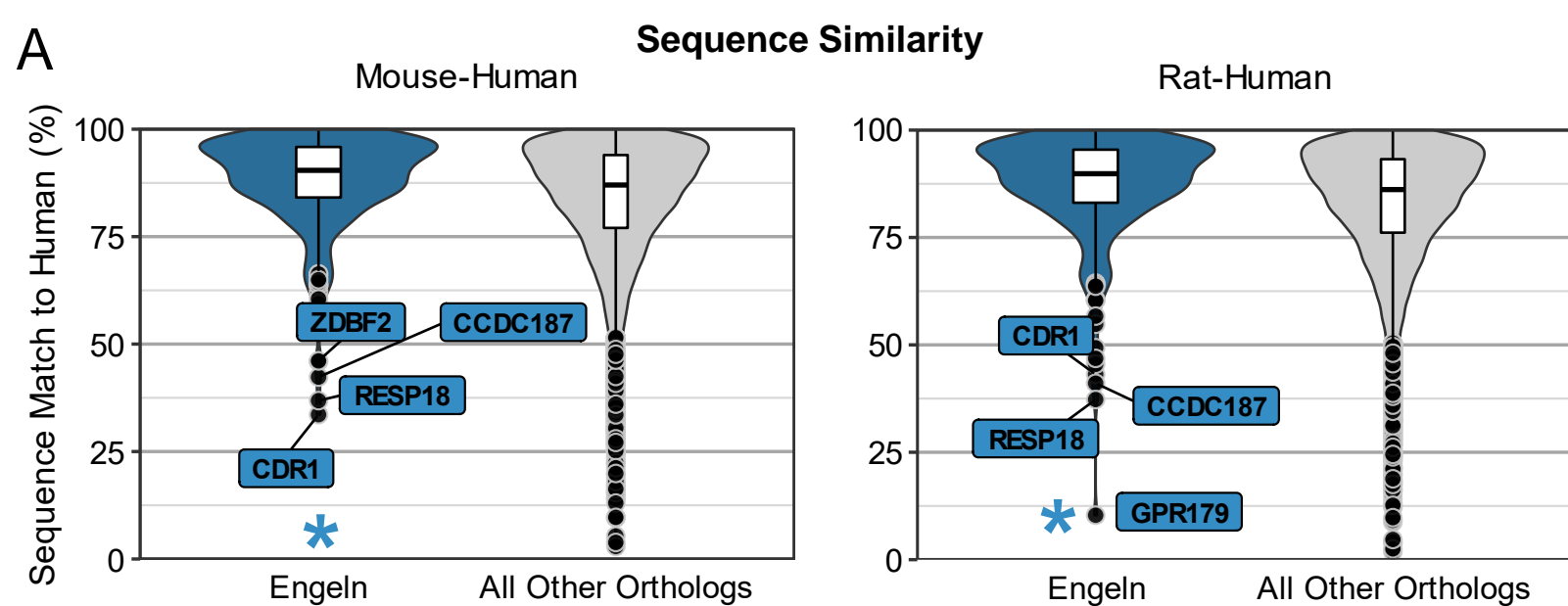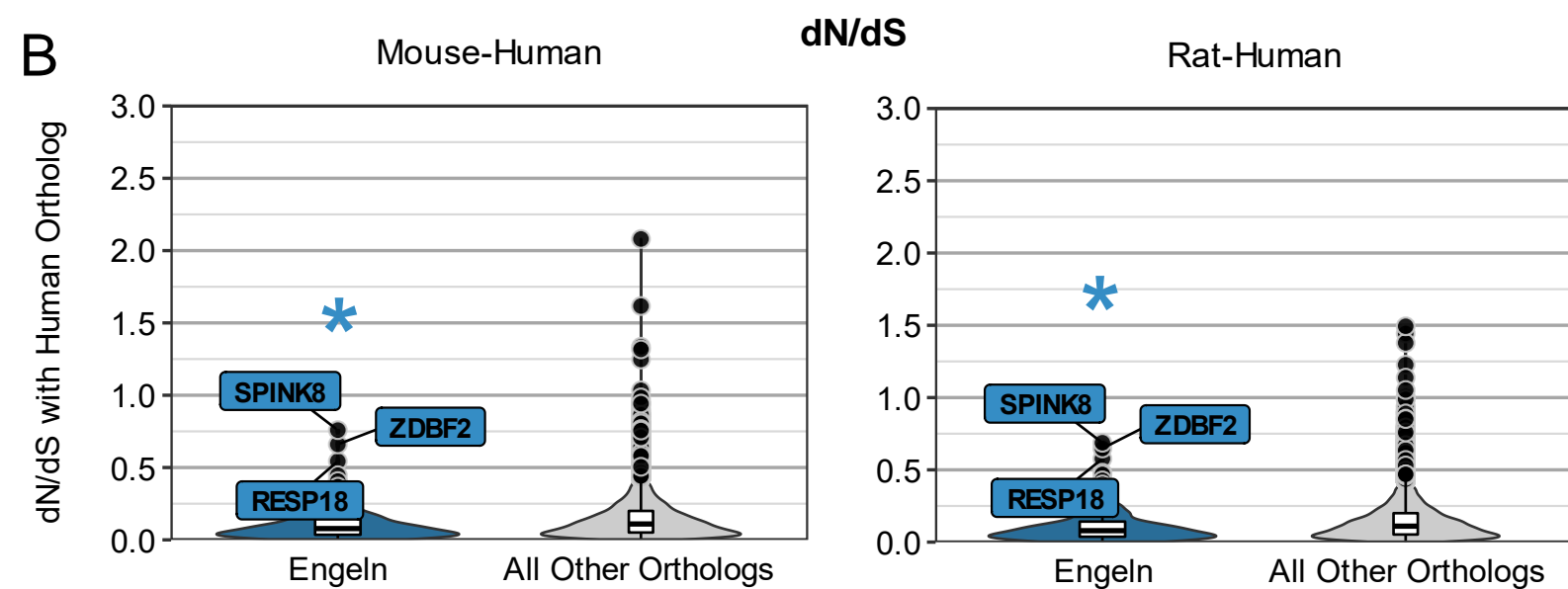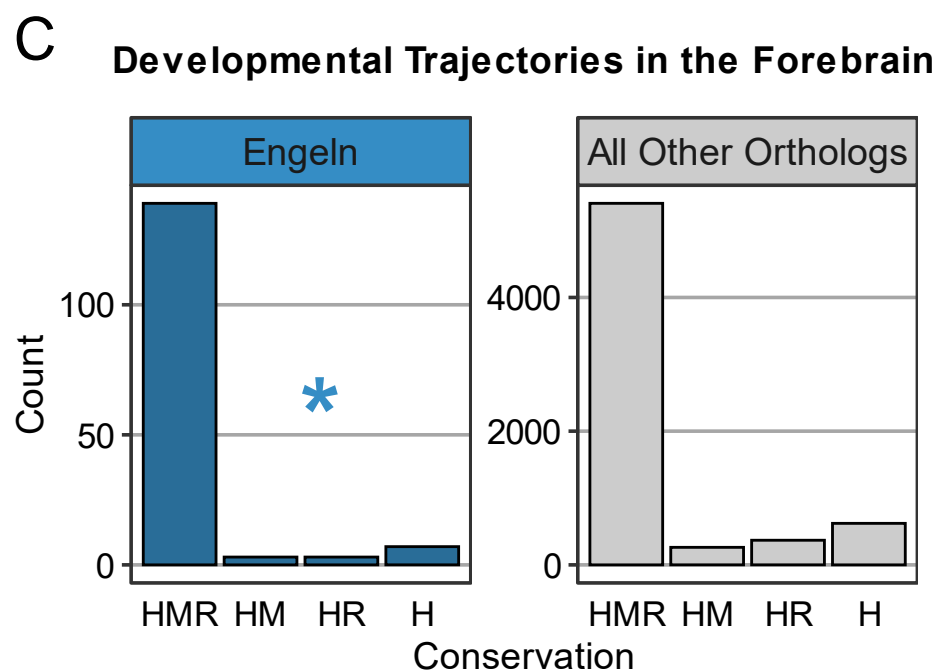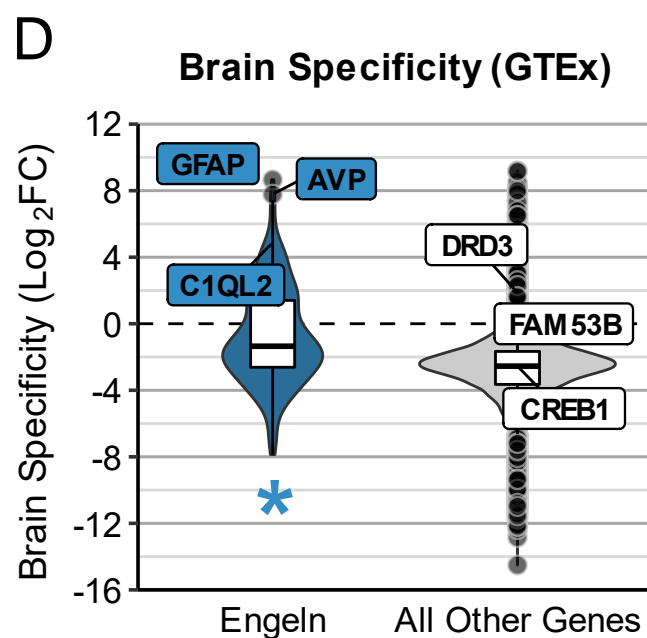

Supplement: jkad143_Supplementary_Data [file jkad143_supplementary_data.zip › Figure_S4_-_FINAL_G3-2022-404013.pdf]
